# Malaria in Tunisian Military Personnel after Returning from External Operation

**DOI:** 10.1155/2013/359192

**Published:** 2013-05-23

**Authors:** Faïda Ajili, Riadh Battikh, Janet Laabidi, Rim Abid, Najeh Bousetta, Bouthaina Jemli, Nadia Ben abdelhafidh, Louzir Bassem, Saadia Gargouri, Salah Othmani

**Affiliations:** ^1^Department of Internal Medicine, Military Hospital of Tunis, 1008 Montfleury, Tunisia; ^2^Department of Parasitology, Military Hospital of Tunis, 1008 Montfleury, Tunisia

## Abstract

*Introduction*. Malaria had been eliminated in Tunisia since 1979, but there are currently 40 to 50 imported cases annually. Soldiers are no exception as the incidence of imported malaria is increasing in Tunisian military personnel after returning from malaria-endemic area, often in Sub-Saharan Africa. *Methods*. We retrospectively analyzed the clinical and biological presentations, treatment, and outcomes of 37 Tunisian military personnel hospitalized at the Department of Internal Medicine, the Military Hospital of Tunis, between January 1993 and January 2011, for imported malaria. The clinical and laboratory features were obtained from the medical records and a questionnaire was filled by the patients about the compliance of malaria prophylaxis. *Results*. Thirty-seven male patients, with a mean age of 41 years, were treated for malaria infection. Twenty-two were due to *Plasmodium falciparum*. The outcome was favourable for all patients, despite two severe access. The long-term use of chemoprophylaxis has been adopted by only 21 (51%) of expatriate military for daily stresses. Moreover, poor adherence was found in 32 patients. *Conclusion*. The risk of acquiring malaria infection in Tunisian military personnel can largely be prevented by the regular use of chemoprophylactic drugs combined with protective measures against mosquito bites.

## 1. Introduction

Malaria is one of the most widespread infectious diseases of our time. According to the latest WHO estimates, there were about 219 million cases of malaria in 2010 and an estimated 660 000 deaths. Africa is the most affected continent: about 90% of all malaria deaths occur there. 

The last few years were marked by an increasing number of imported malaria supported by the increasing number of international travel in association with the important influx of immigrants from malaria-endemic countries especially from the Sub-Saharan Africa [1]. Malaria burden is difficult to estimate, especially in low-income countries where data collection and reporting quality are poor. Data emerging from WHO reports just estimate malaria incidence and mortality, reporting malarial cases and malarial death from the different WHO regions, collected by ministries of health of different countries. These data do not reflect the real incidence in the general population [2]. In Tunisia, we declare currently between 40 and 50 annual cases of imported malaria. *Plasmodium falciparum* is the origin of the majority of the cases [3, 4]. Soldiers are no exception, as malaria represents a common risk threatening sometimes the vital prognosis in military dealing with external operation. The returned infected militaries are a source of parasite and may lead to the reappearance of malaria in countries where it was previously eradicated. It essentially reflects a misapplication of prophylactic measures. 

The aim of this paper is to review the literature about imported malaria in soldiers and to assess the compliance of malaria prophylaxis among the soldiers in our military hospital in the department of internal medicine during the period from January 1993 to January 2011.

## 2. Patients and Methods

The malaria prophylaxis was based on mefloquine at a dose of 250 1 tab/week began a week before departure and continued during the stay and 4 weeks after return or doxycycline 200 at a dose of 1 tablet/day started on the day of departure and continued during the stay and 4 weeks after return. The soldiers were educated to respect the recommendations for malaria prophylaxis that is the protection against mosquito bites (mosquito nets, repellents, impregnated clothing and coils). After returning from a mission in endemic countries (Rwanda, Cambodia, the Democratic Republic of Congo, Cote d'Ivoire), we detected 37 cases of malaria. A questionnaire was filled by all the patients and they gave notification whether they stopped prophylaxis or not, their adherence to the treatment, and the reasons of the noncompliance.

The diagnosis of malaria was so mentioned in front of an infectious syndrome confirmed by testing gout thick. All patients underwent blood samples including parasitological test (thick blood smear), a biological assessment (hematological blood sample, liver and kidney blood tests, balance inflammatory), and clinical monitoring (temperature, neurological status). 

## 3. Results

The patients were all male with an average age of 41 years. The long-term use of chemoprophylaxis has been adopted by only 21 (51%) of expatriate military for daily stresses. Moreover, poor adherence was found in the questionnaire of 32 patients. The fears were iatrogenic risk of impotence infertility in all the non-compliant soldiers. This poor compliance of prophylaxis resulted in 37 malaria cases in the Tunisian Armed Forces after returning from a mission in endemic countries.

Patients were received initially in the emergency department after returning from the malaria-endemic area. All patients had a thin/thick blood smear in the parasitological laboratory of our hospital. No confirmation was made elsewhere. This test allowed the diagnosis of malaria in all the cases. No patient was treated without confirmation by this test. 

The patients were then transferred to the internal medicine department. Clinically, the malaria was dominated by a high fever in all patients. Twenty patients (54%) had nausea and/or vomiting, headache and arthralgia. Thrombocytopenia was constant. Six patients (16%) had severe thrombocytopenia (platelets < 30 × 10^3^/mm^3^). Hepatic cytolysis was found in 75% of cases and a biological inflammatory syndrome in 90% of cases with a mean CRP = 80 mg/L, fibrinogen average = 6 g/L, and an average sedimentation rate = 60 mm in H1. 

The majority of patients (35 cases, 94%) showed no admission severity criteria adopted by WHO [1] but 2 patients who developed cerebral malaria (one case) and severe renal failure with acute tubular necrosis (one case). 

*Plasmodium (P.) falciparum* was the most frequent species (22 cases, 60%) seen in patients returned from Rwanda and Democratic Republic of Cong, followed by *P. ovalae* (10 cases, 27%) returned from Democratic Republic of Congo, *P. vivax* (4 cases, 10%) in Cote D'Ivoire, and *P. malariae* (a case, 2%) in Cambodia.

Parasitemia ranged between 3 and 6%. It exceeded 5% in 4 patients. All patients received a treatment with mefloquine 250 divided into 3 doses at 8 h (3 then 2 then 1 tablet) or artemether + lumefantrine (Coartem) 6 taken in total over 3 days at the following times: H0, H8, H24, H36, H48, and H60. Under malaria treatment, the clinical course was quickly favorable with a thermal defervescence and a disappearance of symptoms in less than four days in 35 cases (94%). There were no deaths among the patients included in the study. Side effects of malaria treatment were reported in 14 patients (38%). They were mostly digestive disorders such as nausea vomiting, diarrhea, dizziness, or rash. We have noted no cases of relapses in our series.

## 4. Discussion

Most of the published studies have assessed the noncompliance after returning from malaria-endemic areas, either through questionnaires filled in by travelers [5–7] or sick patients or through prophylaxis plasma concentration in sick patients. Military in mission in an endemic country, prophylactic treatment noncompliance was estimated between 63.4 and 54.7% in different series [8–10]. 

McCarthy and Coyle [11] had analyzed the effect of malaria chemoprophylaxis drug use in potential travelers. The authors concluded that potential travelers were more tolerant of taking prophylaxis if associated with no or mild adverse events and least tolerant of mild squeals from malaria and severe drug related events. 

Our study had evaluated the compliance with malaria prophylaxis of military travelers by a questionnaire and showed their noncompliance or poor adherence to this treatment. Such preventative measures in our military even if they were poor, they have participated in the development of these clinical forms less serious in our series. Soldiers were not aware of the severity of the malaria disease and even 20 of them declared their noncompliance to the malaria prophylaxis; others may be hiding the truth regarding their compliance. 

The limits of our study were the missing of the practice of blood sampling regarding the concentration of doxycycline or mefloquine, the retrospective character, and the monocentric study as some asymptomatic soldiers may probably be detected for malaria elsewhere with a great delay.

According to WHO, the *malaria elimination* terminology should be adopted when referring to the interruption of local mosquito-borne malaria transmission and the reduction to zero of the incidence of infection caused by human malaria parasites in a defined geographical area as a result of deliberate efforts, but continued measures to prevent reestablishment of transmission are required. 

In Tunisia, malaria was eliminated since 1979; malaria remains topical in Tunisia because of the persistence of anopheles and the coexistence of a potential reservoir of parasites consisting of imported cases of the disease. From 1999 to 2006, 98 cases of imported malaria were diagnosed at the Pasteur Institute of Tunis, which lists about 30% of national cases [3]. No military studies had been published in this field.

There are four types of human malaria: *P. falciparum, P. vivax, P. malariae,*  and  *P. ovale* [12]. In our series, *P. falciparum* was the most frequent species (22 cases, 60%), followed by *P. ovalae* (10 cases, 27%), *P. vivax* (4 cases, 10%), and *P. malariae* (a case, 2%). *P. falciparum *and* P. vivax* are the most common but *P. falciparum* is the most fatal if not treated within 24 hours [13, 14].

In *P. vivax *and *P. ovale *infections, patients having recovered from the first episode of illness may suffer several additional attacks after months or even years without symptoms. Relapses occur because *P. vivax *and *P. ovale *have dormant liver stage parasites that may reactivate [15]. We have noted no cases of relapses in our series.

Concerning our military, they have performed their mission in countries where *P. falciparum* was the dominant species. Mean time to diagnosis of malaria was relatively short 24 hours (6 H–4 days) since all soldiers returning from a mission in an endemic country were screened and this explains also the rarity of severe cases in our series and the good response to the treatment. Because of parasite resistance to antimalarial drugs conventionally used, monotherapy is now banned in the treatment of uncomplicated *P. falciparum*. Chloroquine has been for nearly 40 years, the first-line drug effective, easy to use, and inexpensive that permitted the control of malaria and its mortality. The emergence of resistance and the extension of *Plasmodium falciparum* and *P. vivax* to this molecule are sometimes multiplied by a factor of five and more, the malaria mortality [16]. Chloroquine has lost its place in the arsenal therapeutic or prophylactic against *P. falciparum* [15, 17]. The best available treatment, particularly for malaria *P. falciparum*, is a combination drug including artemisinin (ACT) [10, 11]. Tunisia adopted the combination therapy recommended by WHO to prevent resistance [4]. 

## 5. Conclusion

The noncompliance of the of preventive measures, mainly, chemoprophylaxis during the stay and return of soldiers from malaria-endemic areas, is partially the origin of these cases of malaria. Awareness of the military and a strengthening of their health education before and during the stay are needed to reduce the incidence of this infection which may involves serious life-threatening in patients.
